# Ultraprocessed Food Intake, Cognition, and Executive Function in Adults: A Systematic Review

**DOI:** 10.3390/nu18091361

**Published:** 2026-04-25

**Authors:** Marina Wöbbeking-Sánchez, María Elena Chávez-Hernández, Lizbeth De La Torre, Silvia Wöbbeking-Sánchez, Alba Villasán-Rueda, Octavio Salvador-Ginez, Luis Miguel Rodríguez-Serrano

**Affiliations:** 1 Facultad de Psicología, Universidad de Salamanca, 37005 Salamanca, Spain; mwobbeking@usal.es; 2 Facultad de Psicología, Universidad Anáhuac México, Huixquilucan 52786, Mexico; 3 Facultad de Psicología, Universidad Pontificia de Salamanca, 37002 Salamanca, Spain; ldelatorrelo@upsa.es; 4 Facultad de Ciencias de la Salud, Universidad Internacional de la Rioja, 26006 Logrono, Spain; swobbekingsa@upsa.es (S.W.-S.); alba.villasan@unir.net (A.V.-R.); 5 División de Estudios de Posgrado e Investigación, Facultad de Psicología, Universidad Nacional Autónoma de México, Mexico City 04510, Mexico; osginez@psicologia.unam.mx

**Keywords:** ultraprocessed food, cognitive function, executive function, adults, NOVA

## Abstract

**Introduction**: This systematic review examines the association between ultraprocessed food (UPF) intake and cognitive and executive function in adults. Given the global rise in overweight and obesity and the increasing consumption of UPFs, understanding their potential impact on brain health is of growing importance. **Method**: A comprehensive literature search was conducted in PubMed, EBSCO, and Scopus databases following PRISMA guidelines. Fourteen studies met inclusion criteria, encompassing cross-sectional, longitudinal, and experimental designs. Risk of bias was assessed using the National Institutes of Health Quality Assessment Tool. **Results**: The majority of studies (78.5%) reported a significant association between higher UPF consumption and poorer cognitive outcomes, including deficits in memory, executive function, and global cognition. Longitudinal studies consistently demonstrated that increased UPF intake is linked to accelerated cognitive decline and a higher risk of mild cognitive impairment and dementia, particularly in middle-aged and older adults. In contrast, cross-sectional findings were more heterogeneous, and evidence in younger populations remains limited and inconclusive. **Conclusions**: Overall, the findings suggest that high UPF consumption may be a modifiable risk factor for cognitive decline. However, methodological variability and the predominance of observational studies highlight the need for further longitudinal and experimental research to clarify causal mechanisms.

## 1. Introduction

Overweight and obesity among adults represent a major global public health issue, associated with increased morbidity, mortality, and healthcare costs [1,2]. In 2022, over 1 billion people worldwide were classified as obese, accounting for 13% of the global population, and prevalence rates have tripled since 1975 [3]. Projections indicate that by 2035, approximately 25% of the global population will be affected, rising to 3.8 billion adults with overweight or obesity by 2050 [3,4]. Among the contributing factors, the consumption of ultraprocessed foods (UPFs) has been identified as a significant determinant [5,6].

UPFs are characterized by a high degree of industrial processing, the addition of additives, and high levels of added sugars, saturated fats, and sodium, along with lower nutritional quality compared to fresh or minimally processed foods [7]. The concept of UPFs was introduced through the NOVA classification system, which categorizes foods into four groups based on the extent and purpose of processing: (1) unprocessed or minimally processed foods, (2) processed culinary ingredients, (3) processed foods, and (4) ultraprocessed foods [8,9,10,11]. This framework has been widely used to better understand the role of industrial food systems in shaping dietary patterns and health outcomes [12,13].

Consumption of UPFs has increased markedly in recent decades, becoming a common, accessible, and appealing component of diets in many countries [9]. A growing body of epidemiological evidence has linked higher UPF intake to obesity and metabolic and cardiovascular diseases, highlighting their relevance as a public health concern [14,15]. More recently, attention has extended to their potential impact on brain health and cognitive function [16].

Cognitive function encompasses processes such as information processing, memory, attention, executive function (including planning, inhibition, and cognitive flexibility), and processing speed [17]. It is a key determinant of an individual’s functioning across the lifespan [18]. Emerging evidence suggests that diet quality influences not only physical health but also cognitive performance [19]. In this context, higher consumption of UPFs has been associated with an increased risk of dementia and other neurodegenerative disorders, including Parkinson’s disease and multiple sclerosis [20,21,22].

Given the increasing consumption of UPFs and their potential impact on both physical and cognitive health, it is important to synthesize the available evidence on this topic. Therefore, the present systematic review aims to evaluate the effects of UPF intake on cognitive and executive function in adults aged 18 years and older. In this regard, it is important to note that the neurobiological impact of UPF could be detectable early in the adult lifespan. Given that these effects are cumulative throughout adult life, they may emerge as subtle associations in young adults and become more evident in later stages (middle and/or older adults). Therefore, the present systematic review included studies that sampled adults starting at 18 years of age to evaluate these associations throughout the full adult life, detecting both early and long-term cumulative outcomes.

## 2. Materials and Methods

### 2.1. Search Strategy

The research question for the present systematic review followed the PICO format with the following elements: population: adults 18 years or older; intervention: UPF intake; comparator: non-UPF intake. Low, medium, or high consumption of UPF; outcome: performance in cognitive and executive function tasks, including neuropsychological tests.

PubMed, EBSCO, and SCOPUS databases were searched in the title and abstract domains using the following key terms: ultraprocessed food intake, processed food intake, cognitive function, executive function, and adults 18 years or older. Databases were searched for articles in English published from 2000, with no search end date restrictions. Inclusion criteria for articles are observational, experimental, randomized controlled trials, non-randomized, longitudinal, cross-sectional, quantitative, including adult samples (18 years or older), and participants with normal cognition. Studies not in English, with participants under 18 years old, with cognitive impairment, or of qualitative design were excluded. These descriptors were searched in the title and abstract fields. The present systematic review was registered in PROSPERO with ID CRD420251249590. Additionally, the PRISMA checklist can be consulted in the Supplementary Materials section.

The studies’ search and extraction was conducted by three authors (R-S, C-H, and S-G) and revised by four authors (W-S, D-L, W-S, and V-R). The PRISMA method was followed for the identification, extraction, and screening of papers included in the present systematic review. Risk of bias was assessed for the final sample of studies included by three reviewers (R-S, C-H, and S-G) and revised by four authors (W-S, D-L, W-S, and V-R). The Quality Assessment Tool for Observational Cohort and Cross-Sectional Studies [23] (accessed on 1 February 2026) was selected for this analysis, which consists of fourteen questions:

Q1: Was the research question or objective in this paper clearly stated?

Q2: Was the study population clearly specified and defined?

Q3: Was the participation rate of eligible persons at least 50%?

Q4: Were all the subjects selected or recruited from the same or similar populations (including the same time period)? Were inclusion and exclusion criteria for being in the study prespecified and applied uniformly to all participants?

Q5: Were a sample size justification, power description, or variance and effect estimates provided?

Q6: For the analyses in this paper, were the exposure(s) of interest measured prior to the outcome(s) being measured?

Q7: Was the timeframe sufficient so that one could reasonably expect to see an association between exposure and outcome if it existed?

Q8: For exposures that can vary in amount or level, did the study examine different levels of the exposure as related to the outcome (e.g., categories of exposure, or exposure measured as continuous variable)?

Q9: Were the exposure measures (independent variables) clearly defined, valid, reliable, and implemented consistently across all study participants?

Q10: Was the exposure(s) assessed more than once over time?

Q11: Were the outcome measures (dependent variables) clearly defined, valid, reliable, and implemented consistently across all study participants?

Q12: Were the outcome assessors blinded to the exposure status of participants?

Q13: Was loss to follow-up after baseline 20% or less?

Q14: Were key potential confounding variables measured and adjusted statistically for their impact on the relationship between exposure(s) and outcome(s)?

### 2.2. Data Extraction and Risk-of-Bias Assessment

PRISMA guidelines were followed for identifying and extracting the articles for this systematic review. The search for and extraction of articles were performed by three authors (R-S, C-H, and S-G). Title and abstract screening, as well as full-text screening, were made by consensus of all authors. Additionally, the risk of bias was assessed by three reviewers (R-S, C-H, and S-G) using the National Institute of Health (NIH) Quality Assessment Tool for Observational Cohort and Cross-Sectional Studies and reviewed by four authors (W-S, D-L, W-S, and V-R).

## 3. Results

### 3.1. Search Results

**Figure 1:**
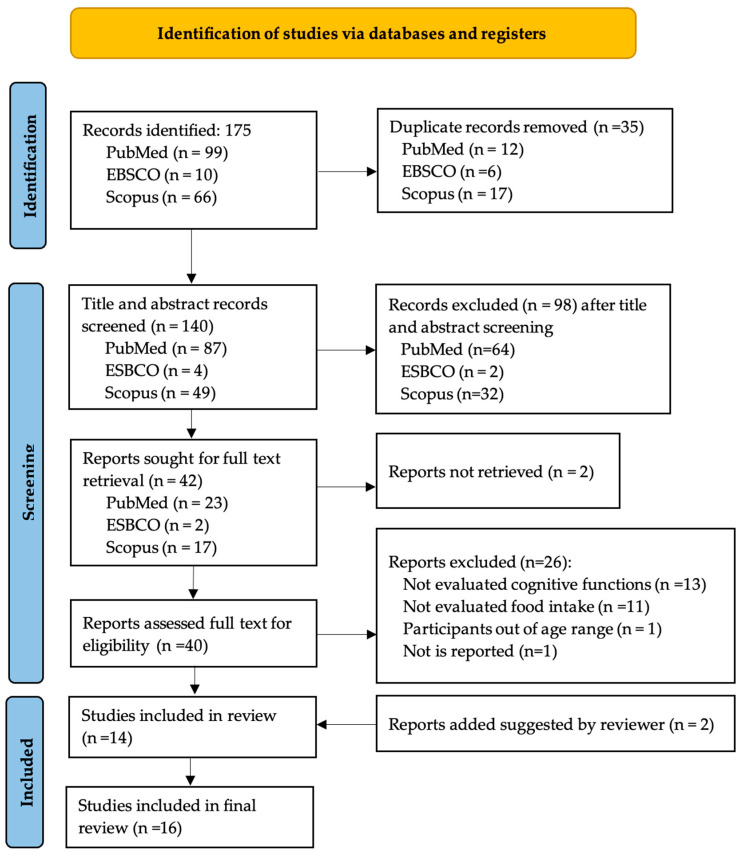
Flow diagram of study selection.

PubMed, Scopus and EBSCO databases search resulted in 175 studies identified, of which 35 were removed on account of being duplicates. Title and abstract screening resulted in 98 studies being removed and 2 articles not retrieved for full text screening. During full text screening, 26 studies were removed, resulting in a final sample of 14 studies included. Additionally, two studies were included in the paper’s reviewing process, resulting in a final sample of 16 studies. Figure 1 shows the PRISMA flowchart for the selection of studies included in the final sample.

Regarding study designs, five included studies (31.3%) made a cross-sectional analysis of data extracted from a longitudinal study, five studies (31.3%) were of prospective cohort design, three studies (18.8%) were of cross sectional design, one was of longitudinal observational design (6.3%), and two were of experimental design (12.3%), one was a randomized, crossover, proof-of-concept trial, and one was a randomized controlled trial.

For UPF intake reports, thirteen studies included a food frequency questionnaire (81.3%), one a Lifetime Diet Questionnaire, one a 24 h recall, and one an UPF diet menu condition. Furthermore, five studies (43.8%) used the NOVA classification system to identify UPF, while eight studies (56.2%) identified dietary patterns through principal components analysis (PCA).

### 3.2. Quality Assessment and Risk of Bias

Table 1 shows the results from the risk-of-bias assessment, performed using the NIH Quality Assessment Tool for Observational Cohort and Cross-Sectional Studies [23] (accessed on 1 February 2026).

### 3.3. Association Between UPF Intake, Cognition and Executive Function

Results from included studies are presented in Table 2. Thirteen studies included in the final sample report a significant association between higher UPF consumption and cognitive and executive function problems (81.3%). On the other hand, three studies did not find a significant association (18.7%). It is important to note that studies that did not find a significant correlation were of cross-sectional design.

### 3.4. The Association Between UPF Intake, Cognition and Executive Function in Different Age Groups

Studies included in the final sample present different participant age groups. Twelve studies included older adult participants (75%). Of these, ten followed a longitudinal design and report a significant association where higher adherence to a Western diet and UPF intake pattern is significantly associated with increased risk of cognitive impairment, cognitive loss, prevalence of mild cognitive impairment, and lower performance in executive function tasks. On the other hand, two studies of cross-sectional design did not find a significant association between UPF intake and cognitive test results.

Two studies (12.5%) included middle-aged adult participants, reporting that higher rates of UPF intake is associated with higher odds of cognitive deficits [24], where consuming more than 19% of daily calories from UPF results in faster rates in cognitive and executive function decline [25].

Finally, two studies with young adult participants (12.5%) show heterogeneous results. In an observational cross-sectional study, where participants self-reported UPF intake and global executive function problems, no significant association was found [26]. On the other hand, a randomized, crossover, proof-of-concept trial found an association between higher inhibitory control and lower total energy intake in an ad libitum buffet meal of the UPF diet condition [27].

**Table 1:** Risk-of-bias assessment using NIH Quality Assessment of Observational Cohort and Cross-Sectional Studies.

Study	Q1	Q2	Q3	Q4	Q5	Q6	Q7	Q8	Q9	Q10	Q11	Q12	Q13	Q14
Akbaraly et al., 2009 [24]	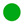	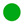	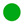	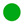	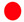	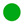	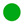	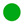	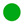	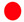	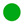	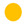	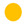	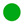
Bhave et al., 2024 [28]	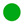	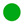	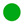	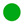	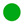	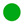	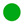	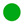	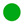	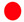	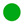	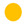	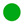	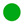
Cardoso et al., 2022 [29]	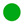	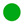	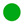	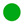	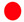	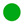	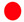	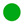	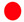	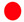	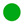	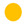	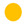	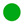
Chávez-Hernández et al., 2025 [26]	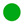	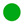	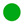	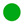	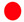	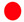	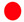	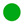	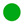	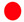	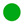	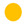	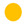	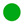
Corley et al., 2020 [30]	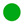	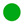	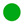	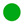	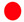	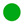	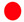	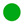	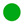	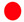	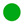	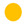	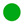	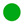
Fu et al., 2022 [31]	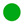	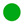	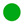	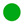	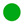	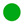	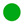	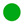	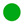	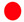	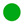	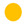	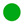	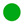
Gomes Gonçalves et al., 2023 [25]	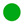	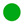	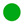	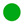	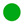	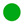	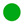	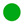	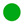	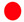	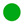	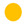	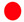	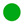
Hosking et al., 2014 [32]	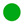	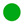	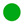	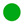	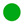	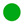	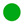	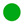	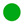	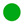	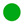	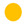	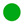	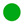
Lee et al., 2025 [33]	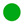	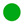	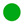	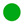	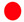	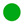	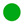	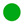	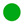	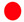	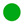	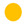	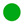	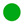
Mumme et al., 2022 [34]	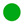	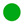	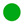	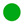	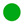	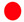	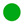	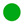	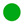	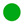	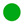	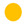	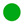	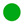
Muñoz-García et al., 2022 [35]	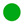	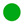	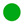	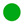	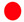	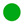	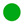	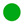	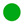	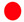	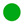	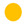	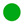	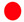
Parrott et al., 2013 [36]	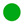	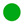	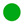	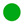	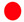	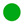	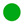	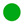	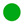	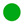	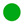	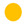	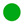	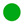
Pearson et al., 2016 [37]	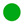	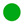	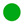	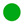	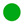	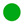	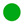	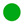	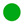	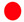	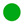	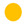	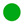	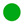
Rego et al., 2026 [38]	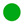	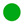	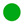	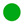	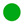	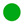	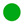	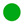	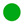	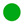	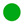	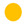	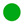	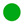
Seago et al., 2025 [39]	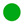	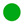	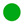	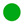	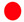	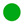	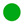	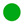	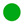	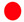	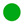	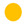	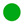	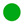
Torres et al., 2012 [40]	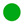	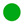	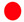	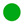	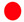	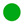	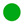	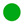	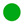	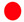	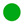	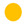	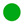	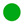

Note: See Section 2 for the full definition of each quality assessment question. low risk of bias (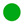); high risk of bias (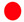); unclear risk of bias (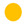).

**Table 2:** Descriptive results from included studies evaluating UPF intake and cognitive function in adults.

Authors and Type of Study	Longitudinal Study and Timepoint	Sample Characteristics	UPF Measure	UPF Determination	Cognitive Measure	Main Results
Akbaraly et al., 2009 [24]. CSA	Whitehall II study. Phase 7 data (2002–2004).	4693 participants Male and female 35–55 years	127-item FFQ	Two dietary patterns identified through PCA: (1) whole food pattern; (2) processed food pattern	Short-term memory. Verbal and mathematical reasoning. Word recognition and comprehension. Verbal fluency.	Processed food pattern higher odds of cognitive deficits than whole food pattern
Bhave et al., 2024 [28]. POC	REasons for Geographic And Racial Differences in Stroke (REGARDS) study	14,175 participants (cognitive impairment cohort). Male and female 45 years or older at 2003–2007.	Block 1998 FFQ	NOVA classification system: NOVA 4 ultraprocessed foods	Memory and fluency.	NOVA 4 consumption associated with increased risk of cognitive impairment
Cardoso et al., 2022 [29]. CS	N/A National Health and Nutrition Examination Survey (NHANES)	2713 participants. National Health and Nutrition Examination Survey (NHANES) Male and female. Mean age 69.1 years.	Two, non-consecutive 24 h dietary recalls	NOVA	Consortium to Establish a Registry for Alzheimer’s Disease (CERAD). Word Learning test: Memory. AFT: Categorical verbal fluency/ executive function. Digit Symbol Substitution test: Processing speed, sustained attention, and working memory.	No significant associations between the UPF intake and cognitive test results
Chávez-Hernández et al., 2025 [26]. OCS	N/A	265 participants. Male and female. Mean age 19.47 years old.	Ultraprocessed food frequency questionnaire: List of foods classified as UP according to the NOVA classification	NOVA	WEBEXEC Questionnaire: Global perception of executive function problems	No significant association between UPF intake and EF problems
Corley et al., 2020 [30]. CSA	Lothian Birth Cohort 1936 (LBC1936) study. Wave 4 data (mean age 79 years).	511 participants. Male and female. Mean age 79.3 years.	EPIC-Norfolk FFQ	Two dietary patterns identified through PCA: (1) Mediterranean-style pattern; (2) processed food pattern	Visuospatial ability. Processing speed. Memory. Verbal ability.	Processed food pattern: Associated with lower global cognitive function. Lower scores in visuospatial ability, processing speed and verbal ability.
Fu et al., 2022 [31]. CSA	Tianjin Elderly Nutrition and Cognition Cohort study. Baseline data.	4457 participants. Male and female. Mean age 67.6.	FFQ	Four dietary patterns identified through RRR: (1) vegetarian; (2) animal foods; (3) processed foods; (4) starchy food	Assessment of MCI: Modified version of the Petersen criteria to diagnose MCI: subjective memory complaints, MMSE, absence of dementia, Alzheimer’s disease, psychiatric disorder(s), cerebral damage, or physical diseases leading to cognitive impairment.	Highest processed food pattern associated with prevalence of MCI
Gomes Gonçalves et al., 2023 [25]. PC	Brazilian Longitudinal Study of Adult Health (ELSA-Brasil)	10,775 participants. Male and female. Mean age 51.6 years.	114-item FFQ	NOVA	Memory. Executive function: semantic and phonemic verbal fluency, and Trail-Making Test B.	>19.9% consumption of UPF calories from daily calories compared to consumption ≤19.9% of daily calories: 28% faster rate of global cognitive decline. 25% faster rate of executive function decline.
Hosking et al., 2014 [32]. LRCT	The older people, omega-3, and cognitive health (EPOCH) trial	352 participants Male and female Age range 65–91 years	Lifetime Diet Questionnaire	Twelve dietary patterns identified through Exploratory Factor Analysis	Speed-based cognitive constructs: perceptual speed, psychomotor speed, reasoning speed, simple/choice reaction time, memory scanning, inhibition. Accuracy-based cognitive constructs: working memory; retrieval fluency, short-term memory, reasoning.	Higher consumption of “coffee and high-sugar, high-fat extras” pattern = poorer performance on simple/choice reaction time, working memory, retrieval fluency, short-term memory and reasoning.
Lee et al., 2025 [33] CSA	Health and Retirement Study (HRS). 2013 and 2016 data.	1408 participants. Male and female. Mean age 70.1 years.	61-item FFQ [41] in 2013 Health Care and Nutrition Study (HCNS)	NOVA	Harmonized Cognitive Assessment Protocol: executive function, memory, language, visuospatial, and orientation.	Marginally significant association between higher UPF intake and worse performance in executive function tasks.
Mumme et al., 2022 [34]. CS	N/A	367 participants. Male and female. Mean age 69.7 years.	109-item FFQ	Three dietary patterns identified through PCA: (1) Mediterranean style; (2) Western; (3) prudent.	COMPASS. Five domains: attention and vigilance, executive function, episodic memory, working memory, and spatial memory	No association between dietary pattern and cognitive domains
Muñóz-García et al., 2022 [35]. PC	“Seguimiento Universidad de Navarra” (SUN) project	806 participants. Male and female. Mean age 67 years.	136-item FFQ	Two dietary patterns identified through PCA: (1) Western diet; (2) Mediterranean diet.	Telephone interview for Cognitive Status (TICS). Four cognitive domains: orientation, memory, attention/calculation, and language.	Higher adherence to Western diet pattern associated with greater cognitive loss.
Parrot et al., 2013 [36]. PC	NuAge study (Quebec Longitudinal Study on Nutrition and Successful Aging)	1099 participants. Male and female. Mean age 94.0 years.	78 foods/ food groups FFQ	Two dietary patterns identified through PCA: (1) prudent pattern; (2) Western pattern.	Global cognitive function evaluated with MMMSE.	Adherence to Western pattern associated with worse overall cognitive performance and more cognitive decline
Pearson et al., 2016 [37]. POC	REasons for Geographic And Racial Differences in Stroke (REGARDS) study	18,080 participants. Male and female. Mean age 64.8 years.	Block98 FFQ	Five dietary patterns identified through PCA: (1) convenience, (2) plant-based, (3) sweets/fats, (4) Southern, and (5) alcohol/salads.	SIS: word recall and temporal orientation. Three-test battery: WLL and WLDR to assess learning and memory domains; AFT to assess executive function.	Southern diet pattern (fried food and processed meat) associated with lower scores in learning, memory and executive function.
Rego et al., 2026 [38]. RCP	N/A	27 participants. Male and female. Mean age 22 years.	Two diet menu conditions, matched in typical US diet composition: non-UPF and UPF.	NOVA	Go/ No-Go task: Inhibitory control	Lower energy intake in UPF diet condition associated with higher inhibitory control.
Saego et al., 2025 [39] LOS	Health and Retirement Study (HRS). 2013 and 2014 data.	4750 participants. Male and female. Mean age 69.0 years.	FFQ	NOVA	Langa–Weir Classifications: immediate recall, delayed recall, serial 7s, and backward counting.	No association between total UPF intake and cognitive impairment. Significant association between animal products and sugar- sweetened beverages with higher risk of cognitive impairment.
Torres et al., 2012 [40]. CSA	18-month randomized, double-blind, placebo-controlled clinical trial. Baseline data.	249 participants. Male and female. Mean age 73.4 years.	107-item FFQ	Two dietary patterns identified through PCA: (1) whole food, (2) processed food.	CAMCOG: eleven cognitive functions.	Negative correlation between overall CAMCOG score. Highest consumption of processed food pattern associated with poorer cognitive functioning, and lowest performance in executive function.

Abbreviations: FFQ: food frequency questionnaire; PCA: principal component analysis; RRR: reduced rank regression; CAMCOG: Cambridge Cognitive Examination; WLL: Word List Learning; WLDR: Word List Delayed Recall; AFT: Animal Fluency Test; MMMSE: Modified Mini-Mental State Examination; MMSE: Mini-Mental State Examination; COMPASS: Computerised Mental Performance Assessment System; SIS: Six-Item Screener; N/A: not applicable. Type of study abbreviations: CSA: cross-sectional analysis; POC: prospective observational cohort; OCS: observational cross-sectional; PC: prospective cohort; LRCT: longitudinal Randomized Control Trial; CS: Cross sectional; RCP: randomized, crossover, proof-of-concept trial. LOS: Longitudinal Observational Study.

## 4. Discussion

The aim of this systematic review was to analyze the association between the consumption of UPF and cognitive function in adults 18 years or older, identifying the cognitive domains most affected and exploring possible differences according to age. Overall, evidence from the included studies suggests a consistent negative association between UFP consumption and cognitive performance. Importantly, the magnitude of this association and the domains affected vary between age groups and according to the methodological quality of the studies included, which should be considered when analyzing this relation.

In the present systematic review, studies included with a longitudinal approach found significant associations between UPF diet patterns and cognitive impairment, while studies with a cross-sectional design show inconsistent results when analyzing this association. In this regard, the effects of UPF intake in cognition may not be detectable in cross-sectional studies, given that the effect may be evident only after a prolonged exposure. Therefore, analyzing cumulative exposure over time and progressive changes in longitudinal studies evidences the adverse effects of UPF intake on cognition. Also, this evidences the relevance of a temporal relationship between dietary exposure and cognitive outcome.

Furthermore, longitudinal studies have linked high UPF intake to an increased risk of developing Alzheimer’s disease in middle age and to faster cognitive decline [42]. Generally, longitudinal studies included consistently show that UPF intake has negative effects in cognition, regardless of population age group included. In this regard, it has been reported that UPF intake reduces executive function and attentional control, and that short-term exposure has an effect on craving and reward responsivity, while the effects on cognition are evidenced in longitudinal cohorts [43].

Studies included in the final sample also include different age groups which indicate different results. Most of the studies (ten out of fourteen, 71.4%) in the final sample include older adult populations (≥65 years old), where UPF intake is associated with a greater risk of cognitive decline in longitudinal studies. In this regard, a systematic review revealed that older adults without preexisting conditions who consumed high amounts of UPF had a higher risk of worse performance in cognitive tasks [44]. Furthermore, evidence indicates that pro-inflammatory diet patterns associated with UPF intake increase the risk of cognitive decline, dementia, and neurodegenerative disorders (e. g. Parkinson’s Disease and multiple sclerosis) [45]

In middle-aged adults, evidence consistently shows that UPF intake has negative effects on cognition. For instance, Gomes Gonçalves et al. [25] observed that middle-aged adults with higher percentage of energy from UPF had faster decline in global cognition and executive functions. Furthermore, it has been shown that high UPF intake in middle and older adults is associated with increased risk of incident dementia [22].

Regarding studies with a young adult population, two included studies evaluated the effect of UPF in this population, with heterogeneous results. Chávez-Hernández et al. [26] did not find a significant association between UPF intake and global executive function problems, while Rego et al. [38] report that participants with lower UPF energy intake have higher performance in inhibitory control tasks. Additionally, it has been reported that young adults aged <30 years old consume the highest energy intake from UPF [46].

Regarding the domains evaluated, studies included assess memory domains (e.g., working memory, short-term memory), verbal fluency, executive function (e.g., inhibition), and cognitive impairment and/or function, the latter particularly in studies with older adult participants. Overall, the results suggest that higher consumption of UPF is consistently associated with poorer cognitive performance and more rapid cognitive decline, worse performance in executive functions, and greater risk of dementia [24,25,28,30,32,35,36,37,40]. Furthermore, these results show that UPF, as products of low nutritional value and high energy density, contribute to adverse metabolic profiles and cognitive problems [20,21].

Díaz-Villaseñor et al. [47] suggest that sociocultural factors and nutrition transition have modified eating patterns, adopting Western diet patterns of industrialized products such as UPF. This change in eating patterns preferring UPF include a substantial increase in daily calorie consumption with a low-quality diet and low intake of proteins, fibers, and micronutrients such as potassium, magnesium, zinc, and vitamins C, D, B12, and niacin [48]. Additionally, it has been shown that UPF acts directly or indirectly on the gut microbiome and modifies the gut–brain axis, inducing neuroinflammation, oxidative stress, and neurodegeneration [49,50]. In summary, UPF consumption contributes to persistent inflammation, both in microbiome and the brain, given that these foods are characterized by important micronutrient deficits, therefore affecting brain function and inducing cognitive decline.

## 5. Conclusions

Overweight and obesity in adults are worldwide public health issues. In this regard, the consumption of ultraprocessed foods is considered an important factor in the development of these conditions. Additionally, consumption of these types of foods has been shown to affect cognition and executive function. Therefore, it is crucial to analyze the evidence regarding the effects of ultraprocessed food intake on adult cognitive and executive function. This systematic review evaluates the impact of ultraprocessed food consumption on the cognitive and executive functions of adults aged 18 and over. Synthesizing findings across studies will provide a better understanding of how ultraprocessed food intake impacts cognitive health and may help develop targeted strategies for addressing this public health issue.

Further longitudinal research is required to consistently stablish causality between UPF intake and impairment in cognitive and executive function. However, our findings suggest that reducing UPF intake may represent a practical intervention to reduce the risk of cognitive impairment. Furthermore, these findings provide scientific basis for public health campaigns to promote healthier food choices over UPF, aiding as well in reducing global overweight and obesity indices and preserving cognitive and executive function.

## 6. Limitations

In the present systematic review, some limitations are present. First, we identified a methodological heterogeneity among the included studies, which together with the predominance of observational designs, may affect the consistency of the results and limit causal inference. Second, potential biases in the measurement of dietary intake and cognitive function, and variability in the control of confounding factors, could affect the robustness of the findings. In this regard, studies included present inconsistency in the UPF classification methods, which presents as a limitation (e.g., principal component analysis of FFQ vs. NOVA classification system). However, due to methodological heterogeneity and the predominance of observational studies, further well-designed longitudinal and experimental studies are required to clarify causal mechanisms and critical exposure periods in the consumption of UPF.

## Data Availability

No new data were created or analyzed in this study. Data sharing is not applicable to this article.

## Supplementary Materials

The following supporting information can be downloaded at: https://www.mdpi.com/article/10.3390/nu18091361/s1, PRISMA 2020 Checklist [51].

## Abbreviations

The following abbreviations are used in this manuscript:

UPF	Ultraprocessed foods
FFQ	Food Frequency Questionnaire
PCA	Principal Component Analysis
RRR	reduced rank regression
RCT	Randomized Controlled Trial
CAMCOG	Cambridge Cognitive Examination
WLL	Word List Learning
WLDR	Word List Delayed Recall
AFT	Animal Fluency Test
MMMSE	Modified Mini-Mental State Examination
MMSE	Mini-Mental State Examination
COMPASS	Computerised Mental Performance Assessment System
SIS	Six-Item Screener
N/A	Not applicable
